# Physical Activity Patterns in Normal-Weight and Overweight/Obese Pregnant Women

**DOI:** 10.1371/journal.pone.0166254

**Published:** 2016-11-09

**Authors:** Elisabetta Bacchi, Cecilia Bonin, Maria Elisabetta Zanolin, Francesca Zambotti, Dario Livornese, Silvia Donà, Flavia Tosi, Giulia Baldisser, Tatsiana Ihnatava, Daniela Di Sarra, Enzo Bonora, Paolo Moghetti

**Affiliations:** 1 Endocrinology, Diabetes and Metabolism, Department of Medicine, University and AOUI of Verona, Verona, Italy; 2 Miniinvasive Pelvic Surgery Unit, AOUI of Verona, Verona, Italy; 3 Epidemiology and Medical Statistics, Department of Diagnostic and Public Health, University of Verona, Verona, Italy; University of Tennessee Health Science Center, UNITED STATES

## Abstract

The aims of the present study were to assess the volume of physical activity (PA) throughout pregnancy in normal-weight vs overweight/obese women, and to investigate which factors may predict compliance to PA recommendations in these women throughout gestation. In 236 pregnant women, 177 normal-weight and 59 overweight/obese (median[IQR] BMI 21.2[19.9–22.8] vs 26.5[25.5–29.0] kg/m2, respectively), medical history, anthropometry and clinical data, including glucose tolerance, were recorded. In addition, pre-pregnancy PA was estimated by the Kaiser questionnaire, while total, walking and fitness/sport PA during pregnancy were assessed by the Physical Activity Scale for the Elderly (PASE) modified questionnaire, at 14–16, 24–28 and 30–32 weeks of gestation. PA volume was very low in the first trimester of pregnancy in both groups of women. However, it increased in the second and third trimester in normal-weight, but not in overweight/obese subjects. Higher pre-pregnancy PA was a statistically significant predictor of being physically active (>150 minutes of PA per week) during all trimesters of gestation. In conclusion, physical activity volume is low in pregnant women, especially in overweight/obese subjects. PA volume increases during pregnancy only in normal-weight women. Pre-pregnancy PA is an independent predictor of achieving a PA volume of at least 150 min per week during pregnancy.

## Introduction

The benefits of regular physical activity (PA) during pregnancy are well-documented. Regular exercise is associated with reduced risk of excessive gestational weight gain and of several pregnancy complications, such as gestational diabetes, preeclampsia, preterm birth, deep vein thrombosis, delivery complications, as well as of anxiety and depression during pregnancy [1–4]. Based on this evidence, physical exercise is generally recommended during pregnancy by all scientific societies and regulatory health agencies [5,6]. In particular, the American College of Obstetricians and Gynecologists recommends that all healthy pregnant women perform an exercise program that leads to a goal of moderate-intensity PA for at least 20–30 minutes per day on most or all days of the week, in the absence of specific contraindications [6].

Interestingly, pregnancy is recognised as a unique time, in which women are extremely motivated to implement healthy behavior. However, against this background, several studies have reported that physical exercise practiced during pregnancy is frequently insufficient to assure the benefits of an active lifestyle.

Previous studies, carried out in different countries, have reported low percentages of physically active women during pregnancy. In the United States, Evenson *et al* collected information, through telephone interviews, on leisure time physical activity of 1979 pregnant and 44.657 non-pregnant women, aged 18–44 yr, reporting that only 15.8% of pregnant women vs 26.1% of non-pregnant women were engaged in the recommended PA volume [7]. This figure was even lower in a study from Brazil, where only 4.7% of pregnant women were physically active [8]. In Europe, Walsh *et al* found that 21.5% of 358 Irish healthy pregnant women between 10–24 weeks of gestation met the recommended PA [9]. In a prospective study carried out in the United Kingdom, where participants self-reported PA during pregnancy at 18 and 32 weeks of gestation, the percentage of women engaging in sufficient PA to cause sweating for at least 3h per week was about 48% in both periods of gestation [10]. In these studies, factors affecting compliance to physical activity recommendations were not investigated.

Available information on the changes in PA patterns throughout gestation is very limited. Only a few studies have investigated the PA levels of pregnant women at each trimester of gestation, with mixed results [8,11,12,13,14]. Some studies have found a progressive decrease in PA across trimesters of gestation [8,11,13], whereas other studies have reported higher PA levels at the second trimester than at the first or the third ones [12,14]. Unfortunately, owing to the limitations of studies (in particular the retrospective or cross-sectional nature of most of them), differences in the time points of pregnancy in which data were collected, the heterogeneity in methods used to assess PA volume, and the different characteristics of subjects, any conclusions on these aspects are vague. Again, data on comparison of normal-weight *vs* overweight/obese women, at each trimester of gestation, are also extremely scarce [12].

To address these issues, the present study aimed at assessing PA volume at each trimester of pregnancy in normal-weight *vs* overweight/obese women, and at investigating which factors may predict compliance to PA recommendations in these subjects throughout pregnancy.

## Material and Methods

### Subjects and design

This study was based on a preliminary analysis of data collected from the Trilogy Study, an ongoing longitudinal study in pregnancy, whose primary aim was detecting early predictors of gestational diabetes and preeclampsia. Pregnant women were recruited in this study on a voluntary basis, among those attending a first trimester nuchal translucency screening test at the Unit of Obstetrics and Gynecology of Verona City Hospital (Verona, Italy).

In this study, inclusion criteria were age between 18 and 40 years and Caucasian ethnicity. The exclusion criteria in the Trilogy Study were pre-pregnancy diabetes mellitus and a multiple pregnancy. In addition, considering the specific aim of this sub-project, women who did not fill in all the requested questionnaires on PA and those who presented contraindications to physical activity, as stated by an obstetrician, were also excluded from this study.

At the time this analysis was carried out, 299 consecutive women enrolled in the Trilogy Study had completed the protocol. A total of 50 subjects did not fill in all the questionnaires. Moreover, 13 women (9 normal-weight and 4 overweight/obese) were excluded due to contraindications to physical exercise. In particular, one of these women had blood abnormalities, one amniotitis, three a risk of miscarriage, five early pre-eclampsia, two a fetus with suspected cardiac abnormalities, and one a fetus with intrauterine growth restriction. The 236 subjects included in this analysis did not differ from the whole sample of women recruited in the Trilogy Study in terms of any demographic features, such as age, parity, smoking behavior, alcohol consumption, or level of education.

Women were enrolled at 12±1 weeks of gestation, and information on age, height, pre-pregnancy weight, education level, job, alcohol and cigarette consumption, medical and obstetric history, and pre-pregnancy PA was collected. Data on educational level, job and alcohol consumption were missing in several subjects.

Subsequently, women were examined at 14–16, 24–28 and 30–32 weeks of gestation, collecting at each time additional information on medical history, current PA and work activity, blood pressure and anthropometric data. Blood and urine samples were also collected at all of these time points. In addition, a 2-h 75-g oral glucose tolerance test was carried out at both 14–16 and 24–28 weeks of gestation, and an additional fasting glucose measurement was obtained at 30–32 weeks.

Weight was recorded on an electronic scale (Tanita BWB-800, MA, USA) and height was measured with a Harpenden stadiometer (Holtain Ltd., Crymych Pembs, U.K.). Blood pressure was assessed by an automatic device.

Diagnosis of gestational diabetes mellitus (GDM) was carried out by using the criteria of the International Association of Diabetes and Pregnancy Study Groups (IADPSG) [15]. Information on other pregnancy complications, which were not contraindications to exercise, (e.g. transient infective diseases, thyroid dysfunction, mild hypertension, etc) were collected. The study was approved by the Ethical Committee of the Azienda Ospedaliera Universitaria Integrata of Verona, Verona, Italy. Informed written consent was obtained from all participants before inclusion in the study. All investigations were conducted according to the principles expressed in the Declaration of Helsinki.

### Assessment of physical activity

At 12±1 weeks of gestation women filled in the modified Kaiser physical activity survey (KPAS) [16], a self-administered questionnaire designed to obtain information about women’s physical activity habits before pregnancy. In addition, at 14–16, 24–28 and 30–32 weeks of gestation, women filled in a Physical Activity Scale for the Elderly questionnaire (PASE), as modified for pregnancy, aimed at estimating PA performed during the prior week [17].

The KPAS questionnaire takes about 20 minutes to complete and questions are grouped into four sections, entitled: 1) “Household and Family Care Activities”, which includes caring for children or the elderly, meal preparation, major cleaning, shopping and gardening; 2) “Occupational Activities”, which includes sitting, standing, lifting heavy loads, walking and sweating from exertion; 3) “Active Living Habits”; which includes watching TV, walking and cycling to and from work or school; and 4) “Participation in Sports and Exercise”, which includes frequency and duration of up to three frequently performed sports or exercises. A Total Activity Index is then calculated as follows: (Household/caregiving index*0.25 + Occupational index*0.25 + Active living index*0.25 + sports/exercise index*0.25)*4.

The PASE questionnaire asks for the minute and days *per* week in which walking activity (WPA) or fitness/sport activities (FSPA), such as swimming, cycling, aerobic class, or other similar activities have been performed in the previous week. In addition, it asks for the intensity of exercise (light, moderate or vigorous). Total physical activity (TPA) is then calculated as the sum of WPA and FSPA. Standard Reference Tables were used for estimating intensity of PA in Metabolic equivalents (METs) [18], and total PA volume, expressed in METs *·* hours per week, was then calculated, as the sum of the METs spent in each activities.

Pregnant women who reported to spend at least 150 minutes *per* week in TPA were classified as “active”, otherwise they were classified as “inactive”.

A validation of the modified PASE questionnaire was preliminary assessed in a sample of 28 consecutive pregnant women, referred before the start of the Trilogy Study to the Obstetrics Unit of our Hospital for physiological pregnancy, by comparing the results of this questionnaire and a 7-day activity diary, as done in previous studies to validate other questionnaires [19,20]. Briefly, in this comparison, activity diary records (type of activity, minute and intensity, reported as light, moderate or vigorous) were kept for seven consecutive days. At the end of the 7 days, women filled in the modified PASE questionnaire. The agreement between the PASE questionnaire and the 7-day activity diary was very good, with a Spearman correlation coefficient of rho = 0.98 for minutes per week, rho = 0.93 for day per week, and rho = 0.93 for PA volume (intensity x duration per week).

### Statistical analysis

Data are shown as median and interquartile range (IQR) or as a percentage, as appropriate. Normality of the distribution of the studied variables was assessed by the Shapiro-Wilk test. Characteristics of the normal-weight and overweight/obese pregnant women were compared by Student t-test or Chi-square test, as appropriate. As minutes, days and METs of TPA, WPA and FSPA could not be normalized, non parametric tests were used to compare normal-weight *vs* overweight/obese subjects at each week of gestation (by the Mann-Whitney test), and to analyze their changes within each group (by the Friedman test). When Friedman analysis detected significant changes in PA variables, the Wilcoxon test for paired data was subsequently performed, adjusting results by post-hoc Bonferroni analysis. The Chi-square test was used to compare the percentages of pregnant women who did at least 150 minutes per week of physical activity between normal-weight and overweight/obese pregnant women at each time point of gestation.

Univariate relationships between PA weekly duration and continuous variables were assessed by the Spearman correlation. Associations with categorical variables were assessed by the Chi-square test. Logistic regression analysis was performed to investigate the independent predictors of achieving a PA volume of at least 150 min per week in the whole cohort of pregnant women. In this analysis, independent variables were chosen on the basis of results of univariate relationships and biological plausibility. Selected independent variables were: age, pre-pregnancy BMI, total weight gain during gestation, total pre-pregnancy physical activity, smoking behavior, nulliparous/multiparous status, occurrence of gestational diabetes mellitus, and occurrence of other pregnancy complications.

Tests with P<0.05 were considered statistically significant. Analyses were carried out using STATA version 14 (StataCorp, College Station, Texas, USA). All original data are within S1 Table.

## Results

Of the 236 pregnant women included in the study, 177 were normal-weight and 59 overweight/obese subjects (mean±SD, and range: 21.1±1.9, 16–24.4 kg/m^2^; and 27.8±3.2, 25–40.5 kg/m^2^, respectively in the two subgroups).

Of these women, 35 (14.8%) had gestational diabetes according to IADPSG criteria, 21 (11.9%) among the normal-weight subjects and 14 (23.7%) among the overweight/obese subjects (p = 0.02 between groups).

Table 1 shows the main characteristics of normal-weight and overweight/obese pregnant women included in the study. Blood pressure at first examination, i.e. at 12 weeks of gestation, was higher in overweight/obese women than in normal-weight women, while age and weeks of work during pregnancy were slightly higher in normal-weight subjects. Gestational weight gain, alcohol consumption, educational level, parity and smoking behavior were similar between groups.

**Table 1:** Main characteristics of normal-weight and overweight/obese pregnant women enrolled in the study. Values are median (IQR) or percentage.

Characteristics	Normal Weight Pregnant Women (N = 177)	Overweight/Obese Pregnant Women (N = 59)
**Age**, *years*	34 (31–36)	32 (29–35)†
**Nulliparous**, *% yes*	49.1	53.7
**Pre-pregnancy BMI**, *kg/m*^*2*^	21.2 (19.9–22.8)	26.5 (25.5–29.0)*
**Systolic blood pressure**, *mmHg*	105 (98–115)	110 (104–120)*
**Diastolic blood pressure**, *mmHg*	65 (60–70)	70 (64–75)*
**Smoking behavior**, *% yes*	4.5	5.5
**Any alcohol consumption**, *% yes*	13.9	13.1
**High level of education**, *%*	51.4	66.7
**Weeks of work during pregnancy**, *weeks*	13 (0–32)	0 (0–22)†
**Gestational weight gain**, *kg*	9 (7.4–11.0)	9 (6.2–12.6)
**Gestational Diabetes Mellitus**, % yes	11.9	23.7†
**Pregnancy Complications**, *% yes*	10.0	13.2
**Kaiser (pre-pregnancy) Physical Activity questionnaire**		
**Household and Family Care index**	2.7 (2.3–3.0)	2.7 (2.2–3.0)
**Occupational Index**	2.7 (2.3–3.4)	3.1 (2.3–3.7)
**Active Living Index**	2.7 (2.2–3.5)	2.5 (2.0–3.0)†
**Sport and Exercise Index**	2.5 (1.2–3.7)	1.7 (1.2–3.0)†
**Total Activity Index**	11.0 (9.6–12.1)	10.1 (9.4–11.3)†

^†^p<0.05, and

*p<0.001 between normal-weight and overweight/obese women.

As regards PA patterns before pregnancy, the KPAS total physical activity index and the sub-indices contributing to this figure, particularly the sport/exercise index and the active living index, were lower in overweight/obese than in normal-weight women (Table 1).

Table 2 shows the weekly volumes of PA throughout pregnancy, in terms of total, and of walking and fitness/sport physical activity, as measured by the modified-PASE questionnaire, in normal-weight and overweight/obese pregnant women, at 14–16, 24–28 and 30–32 weeks of gestation.

**Table 2:** Weekly physical activity across gestation in normal weight and overweight/obese pregnant women. Data are expressed as minutes of PA per week, days per week in which PA was performed, and total volume of PA, expressed in METs · hours per week.

	Normal Weight Pregnant Women (N = 177)			*P value*†	Overweight/Obese Pregnant Women (N = 59)			*P value*†	Normal Weight *vs* Overweight/Obese Pregnant Women		
	Weeks of gestation, Median (IQR)				Weeks of gestation, Median (IQR)				Weeks of gestation, P value*		
	14–16	24–28	30–32		14–16	24–28	30–32		14–16	24–28	30–32
**Total Physical Activity per week**											
**Minutes**	60 (0–150) ^a^	120 (40–217)	120 (40–210)	**<0.001**	60 (0–123)	60 (0–127)	60 (0–120)	0.44	0.39	**0.006**	**<0.001**
**Days**	2 (0–5) ^a^	4 (2–6)	4 (2–6)	**<0.001**	2 (0–4)	3 (0–4)	2 (0–4)	0.80	0.48	**0.006**	**0.002**
**PA Volume (METs·h)**	4 (0–8) ^a^	6.7 (2–12)	6 (2–12)	**<0.001**	3 (0–6.8)	3 (0–7.8)	3 (0–6)	0.62	0.36	**0.004**	**<0.001**
**Walking Activity per week**											
**Minutes**	60 (0–120) ^a^	80 (30–180)	70 (0–150)	**0.003**	60 (0–120)	60 (0–115)	45 (0–90)	0.25	0.66	0.087	**0.011**
**Days**	2 (0–4) ^a^	3 (1–5)	3 (0–5)	**0.022**	2 (0–4)	2.5 (0–4)	2 (0–3)	0.84	0.76	0.052	**0.032**
**PAVolume (METs·h)**	3 (0–6.6) ^a^	4.2 (1.5–9.9)	3.7 (0–9)	**0.002**	3 (0–6.1)	3 (0–6)	2.2 (0–4.5)	0.39	0.62	0.063	**0.011**
**Fitness and Sport Activity per week**											
**Minutes**	0 (0–0) ^a^	0 (0–60)	0 (0–60)	**<0.001**	0 (0–0)	0 (0–0)	0 (0–0)	0.48	0.085	0.058	**0.033**
**Days**	0 (0–0) ^a^	0 (0–1)	0 (0–2)	**<0.001**	0 (0–0)	0 (0–0)	0 (0–0)	0.49	0.090	0.082	**0.025**
**PA Volume (METs·h)**	0 (0–0) ^a^	0 (0–4)	0 (0–3)	**<0.001**	0 (0–0)	0 (0–0)	0 (0–0)	0.48	0.081	0.054	**0.033**

* P value obtained by Mann-Whitney test;

^†^ P value obtained by Friedman test;

^a^ P value <0.05 between 14–16 and 24–28 weeks of gestation;

^b^ P value <0.05 between 24–28 and 30–32 weeks of gestation (by Wilcoxon test with Bonferroni correction).

At 14–16 weeks of gestation the two groups of women showed similar patterns of PA, in terms of minutes, days and METs hours *per* week of total, walking and fitness/sport PA. Thereafter, in normal-weight women, minutes, days and METs of PA significantly increased. In particular, in normal-weight women, these volumes increased from 14–16 to 24–28 weeks of gestation and remained substantially stable thereafter. Conversely, these figures did not show any change in overweight/obese subjects (Table 2).

**Fig 1:**
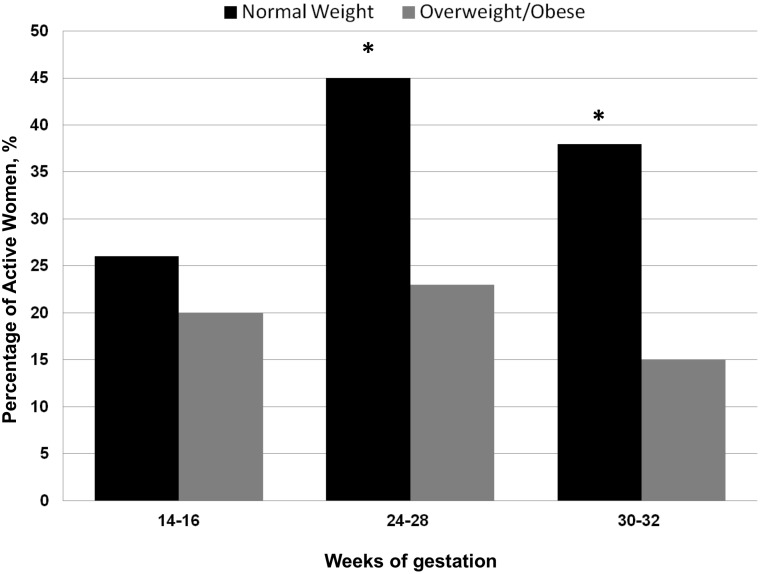
Percentages of women who met the goal of at least 150 minutes of physical activity per week during pregnancy in normal-weight and overweight/obese women, at 14–16, 24–26 and 30–32 weeks of gestation, respectively. * p<0.05 between normal-weight and overweight/obese women (by the Chi-square test).

Fig 1 shows the percentages of normal-weight and overweight/obese women engaged in at least 150 minutes of physical exercise, at 14–16, 24–28 and 30–32 weeks of gestation, respectively. This percentage was similar in the two groups at 14–16 weeks of pregnancy (p = 0.38). However, it was higher in normal-weight than in overweight/obese women at both 24–28 (p = 0.003) and 30–32 weeks (p = 0.001). It is noteworthy that, in the entire cohort of subjects, the percentage of women that reported PA volumes corresponding to standard recommendations throughout pregnancy was 11.8%.

In univariate relationships, weekly duration of PA in each trimester of gestation was associated with pre-pregnancy PA (rho = 0.29, rho = 0.28, and rho = 0.29, respectively in the three trimesters of gestation). Logistic regression analysis was subsequently carried out to investigate the predictors of physical activity (PA>150 minutes *per* week) in these subjects, at 14–16, 24–28, and 30–32 weeks of gestation (Table 3). Interestingly, in the entire cohort of women, physical activity during each trimester of gestation was independently predicted by higher physical activity before pregnancy. In particular, in the first trimester of gestation, physical activity was independently predicted only by this figure (Table 3). However, during the second trimester, lower pre-pregnancy BMI, nulliparous status, and diagnosis of GDM were additional independent predictors of physical activity (Table 3). Finally, during the third trimester of gestation, physical activity was independently predicted by higher pre-pregnancy physical activity, BMI and use of cigarettes (Table 3). Results did not change even after including other independent variables in the model.

**Table 3:** Predictors of achieving the PA volume of at least 150 minutes/week of physical exercise at 14–16, 24–28 and 30–32 weeks of gestation.

Variables	14–16 weeks of gestation (R^2^ = 12%)		24–28 weeks of gestation (R^2^ = 13%)		30–32 weeks of gestation (R^2^ = 11%)	
	OR (95% CI)	P value	OR (95% CI)	P value	OR (95% CI)	P value
**Age**	1.05 (0.96–1.15)	0.274	1.05 (0.96–1.14)	0.229	1.07 (0.98–1.16)	0.110
**Pre-pregnancy BMI**	0.92 (0.82–1.03)	0.133	0.90 (0.82–0.99)	**0.037**	0.90 (0.81–0.99)	**0.048**
**Gestational weight gain**	0.98 (0.89–1.07)	0.719	1.01 (0.93–1.10)	0.708	1.02 (0.93–1.11)	0.595
**Smoking behavior**	3.19 (0.65–15.65)	0.152	2.77 (0.62–12.45)	0.182	4.96 (1.12–21.90)	**0.034**
**Pre-pregnancy total physical activity**	1.57 (1.28–1.93)	**<0.001**	1.41 (1.18–1.67)	**<0.001**	1.44 (1.21–1.73)	**<0.001**
**Nulliparous status**	1.14 (0.56–2.34)	0.704	2.96 (1.57–5.59)	**<0.001**	1.50 (0.78–2.85)	0.216
**Gestational diabetes mellitus**	1.71 (0.66–4.41)	0.266	2.83 (1.16–6.87)	**0.021**	1.15 (0.47–2.85)	0.748
**Pregnancy Complications**	2.63 (0.97–7.11)	0.057	1.58 (0.62–4.04)	0.334	1.52 (0.57–4.00)	0.395

## Discussion

To the best of our knowledge, this is the first prospective study to assess physical activity volume in pregnant women at each trimester of gestation, and compare normal-weight vs overweight/obese women, while also investigating which factors may predict compliance to PA recommendations in different periods of pregnancy. Interestingly, although in the entire cohort of pregnant women only a minority achieved the goal of at least 150 min per week of PA, this fraction was smaller in overweight/obese women than in normal-weight women. In addition, in normal-weight women, total physical activity significantly increased from the first to the second trimester of gestation and remained stable thereafter. Conversely, overweight/obese women did not show any change in PA volume across pregnancy. Finally, being physically active during pregnancy was best predicted, in each trimester of gestation, by higher PA before pregnancy.

Only a few previous studies have assessed PA patterns throughout gestation, with inconsistent findings [8,10,11,12,13,14,21]. These studies have reported either a progressive decrease in PA during pregnancy [8,11], or no change [10], or higher PA levels in the second than in the first or the third trimester of gestation [12,14]. Unfortunately, most studies are limited by the retrospective or the cross-sectional nature of data, and comparisons are constrained by differences in study design, time points of pregnancy, and social and ethnic characteristics of the populations under study.

In a large prospective study, 9889 pregnant women from the UK filled in a questionnaire, at both 18 and 32 weeks of gestation, asking whether they engaged in any regular PA sufficient to cause sweating (defined as strenuous PA) for at least 3h per week and detailed types of self-reported leisure-time PA at 18 weeks [10]. In the whole population, the percentage of physically active women was similar in these two periods of pregnancy. In this study, women with pre-pregnancy obesity were less likely to report brisk walking at 18 weeks, whereas this information was not collected at 32 weeks. However, there were no BMI-related differences in strenuous PA at either 18 or 32 weeks. A study from Nascimento *et al* reported, among 1279 healthy Brazilian women investigated using a questionnaire compiled retrospectively 12–72 hours postpartum, that the percentage of active women was higher in the second than in the first and third trimester of gestation [14]. Renault *et al*, in a cross-sectional study carried out in Danish women who wore a pedometer for at least 5 days and were subdivided according to pre-pregnancy BMI (normal-weight *vs* obese women) and trimester of gestation (first, second or third), found that mean footsteps were higher in normal-weight than in obese women, and, in both BMI groups, at mid-gestation than at early or late gestation [12]. However, Dominigues *et al*, in another cohort of 4471 Brazilian women who, soon after delivery, compiled retrospectively a self-reported questionnaire investigating whether they engaged in some level of leisure time physical activity during pregnancy, reported a progressive decline of the percentage of physically active women, from 10.4% in the first to 6.5% in the third trimester of pregnancy [8]. Similarly, a small study, carried out using both a questionnaire and heart rate monitoring in 23 Swedish normal-weight women, reported a small PA decline between 14 and 32 weeks of gestation [21]. Similar findings were also reported in a study carried out in 305 overweight or obese women who filled in a questionnaire at different time points during pregnancy, and reported a decline in PA between early and middle-late pregnancy [13]. Finally, a small prospective study, aimed at comparing accelerometers and self-reported PA measures and carried out at different time points during pregnancy in a sample of 57 women from the UK, reported a significant decline in PA volume from 12–16 to 34–38 week of gestation [11].

Previous studies in pregnant women have reported large differences in the percentage of physically active women from 5 up to 61% [2,5,8,9,10,22]. These differences may reflect a number of factors, such as the socio-economic and cultural characteristics of the women investigated. Moreover, in these studies PA was assessed by different methods and at different time points during pregnancy. In our study, this percentage increased significantly, from 26% in the first trimester to 45% in the second trimester, in normal-weight women, whereas it increased non-significantly in overweight/obese women, from 15 to 23%, respectively. Our findings in normal-weight subjects are in line with the observation that the optimal time to increase PA during pregnancy is generally after the conclusion of the first trimester, when in most women both fear of pregnancy loss and several pregnancy-induced complaints, such as nausea and asthenia, decline. It is noteworthy that an increase in PA did not occur in overweight/obese subjects, although these women were those who potentially might gain more benefit from increased PA. The predominant modality of PA among our pregnant women was walking in both normal-weight and overweight/obese women, consistent with findings of other studies [7,10,14]. Overall, our data suggest the importance of reinforcing suitable advice and specific interventions for overweight/obese pregnant women, in order to increase PA practice.

In our study, higher pre-pregnancy physical activity was a predictor of physical activity during pregnancy, which is also consistent with previous literature [14]. While this is a confirmatory finding, this paper represents an advance in available knowledge, showing that this is true at each trimester of gestation. This is important information, with a potential impact on healthy policies. In particular, in the entire cohort of women, physical activity during pregnancy was independently predicted at first trimester only by higher pre-pregnancy PA, whereas additional predictors were observed during the second trimester, i.e. lower pre-pregnancy BMI and nulliparous status, and the third trimester, i.e. a higher use of cigarettes. Previous studies reported that the number of previous pregnancies [14] and pre-pregnancy BMI may both affect lifestyle in pregnant women [8,23]. In this regard, women without children at home may have more leisure time for physical exercise, whereas women with a high pre-pregnancy BMI may be expected to continue with a sedentary lifestyle also during pregnancy. The medical recommendation of an active lifestyle in pregnant women after diagnosis of GDM may account for the relationship between this condition and physical activity in the second trimester. Nonetheless, in our study, the observation that a physically active status during late pregnancy was also associated with a higher use of cigarettes is puzzling. We hypothesize that women who smoke during pregnancy may tend to compensate with a healthier lifestyle in other aspects, such as exercise, at least in a cohort of women with quite a high socio-cultural level.

The strengths of this study are the prospective design and the assessment of several clinical, metabolic and physical activity parameters, in both normal-weight and overweight/obese women, at different time points of gestation. Among the main limitations there is the somewhat small sample size. However, it did not prevent us from finding several statistically significant results. Another limitation is the use of self-reported pre-pregnancy weight, and a self-reported questionnaire to assess physical activity. The subjective characteristic of responses, differences in perception of effort and retrieval capacity, and cultural factors may introduce bias in the collection of data. However, objective devices for monitoring PA cannot be used easily in large epidemiological studies and may also have limitations, especially in pregnant women. Notably, the modified PASE questionnaire investigates 7-day periods immediately preceding administration, and has been specifically designed to quantify both duration and intensity of reported activities. In addition, a validation of the questionnaire was carried out in a sample of pregnant women. Finally, we studied a population of Caucasian women with a generally high level of education, precluding the possibility of extrapolating these findings to women with different ethnic and social characteristics.

## Conclusion

In conclusion, PA volume is strikingly low in pregnant women, especially among overweight/obese subjects. PA volume increases during pregnancy in normal-weight, but not in overweight/obese women. Finally, pre-pregnancy physical activity is the best predictor of achieving the goal of at least 150 min per week of PA during all trimesters of pregnancy.

Our data suggest that future interventions, with the goal of increasing physical exercise in pregnant women, should give special attention to overweight/obese subjects, and highlight the crucial role of a primary intervention, when women are still planning a pregnancy. All pregnant women should be evaluated at each trimester of gestation to assess their physical activity volume and the effects of exercise on both maternal and fetal outcome, in order to recommend appropriate changes when necessary. Overall, further work is needed to understand the best way to increase PA levels or instigate behavior changes in normal-weight and especially in overweight/obese pregnant women.

## Supporting Information

S1 Table Original data. (XLS)
